# Template-Assisted Self-Assembly of Conductive Polymer Electrodes for Ionic Electroactive Polymers

**DOI:** 10.3389/fbioe.2020.00837

**Published:** 2020-07-31

**Authors:** Andrew Jo, Clémence Huet, Hani E. Naguib

**Affiliations:** ^1^Department of Chemical Engineering and Applied Chemistry, University of Toronto, Toronto, ON, Canada; ^2^Department of Material Science and Engineering, Polytech Nantes, Nantes, France; ^3^Department of Mechanical and Industrial Engineering, University of Toronto, Toronto, ON, Canada

**Keywords:** self-assembly, electroactive polymer, core shell polymer, low voltage, polyaniline nanofibers, semi-interpenetrating polymer network, ionic liquid-free

## Abstract

Ionic electroactive polymers (ionic EAPs) can greatly aid in biomedical applications where micro-sized actuators are required for delicate procedures. Since these types of actuators generally require platinum or gold metallic electrodes, they tend to be expensive and susceptible to delamination. Previous research has solved this problem by replacing the metallic electrodes with conductive polymers (CP) and forming an interpenetrating polymer network (IPN) between the conductive polymer (CP) and the solid polymer electrolyte (SPE). Since these actuators contain toxic ionic liquids, they are unsuitable for biological applications. In this study, we present a novel and facile method of fabricating a biocompatible and ionic liquid-free actuator that uses semi-IPN to hold the CP and Nafion-based SPE layers together. Surface activated fabrication treatment (SAFT) is applied to the precursor-Nafion membrane in order to convert the sulfonyl fluoride groups on the surface to sulfonate. Through template-assisted self-assembly, the CP electrodes from either polyaniline (PANI) or poly(3,4-ethylenedioxythiophene) (PEDOT) interlock with the surface treated precursor-Nafion membrane so that no delamination can occur. The electrodes growth pattern, interfacial layer’s thickness, and shape can be controlled by adjusting the SAFT concentration and duration.

## Introduction

Conventional actuators are used in minimally invasive surgeries due the improved control and maneuverability given to the surgeon; however, due to its small size, they are expensive to manufacture (Turchetti et al., 2012). Since these devices are made up of multiple rigid parts linked together through a series of joints, a micro-sized actuator can become difficult to assemble and program (Alici, 2018). Instead, electroactive polymers (EAPs) can be used as a cost-effective actuator. This is possible because EAP-based actuators have one continuous body which means that there are less parts needed to be assembled, and it also simplifies the programming of its movements (Alici, 2018). Ionic polymer metal composite (IPMC) actuators are EAPs that have been studied extensively in literature, but these devices require its electrodes to be made with either gold or platinum and therefore are too expensive to mass produce (Bhandari et al., 2012; Lee et al., 2013; Kotal et al., 2016; Rasouli et al., 2017; Hu et al., 2019). Alternatively, conductive polymer (CP) actuators can be used instead. Since these actuators are not constrained to metallic electrodes, they are more affordable, and can produce higher actuation strains (Hu et al., 2019).

Nafion-based solid polymer electrolyte (SPE) with electrodes have been extensively used since Nafion is widely available and is biocompatible (Lee et al., 2013; Kotal et al., 2016; Rasouli et al., 2017). However, many studies have reported that combining Nafion with CP electrodes will result in poor actuations at low voltages (Lee et al., 2013; Vreeland et al., 2015; Rasouli et al., 2017; Hu et al., 2019). One study coated the Nafion surface with carbon nanotubes (CNTs) and carbon black (CB) ink before polymerizing the membrane with CP electrodes, but found this method showed unnoticeable tip displacements below 2.0 V (Rasouli et al., 2017). These devices were also easy to delaminate as the CNT and CB ink are held together through van der Waal forces (Luo et al., 2008). Others have used m-Cresol as an adhesive to bind the polyaniline (PANI) electrodes onto both sides of the Nafion membrane, but this method also showed low tip displacements at low voltages (Lee et al., 2013). They suggested that the low tip displacements observed was due to the actuation of the CP and the Nafion occurring at opposite directions and thus canceling out its overall actuation. In all cases, delamination was a problem and so the Nafion membrane is roughened before attaching the CP electrodes (Liu et al., 2007; Bhandari et al., 2012; Khmelnitskiy et al., 2019; Kim and Kim, 2019).

As noted previously, CP electrodes also have the problem of delamination when attached to a SPE (Bhandari et al., 2012; Lee et al., 2013; Kotal et al., 2016; Rasouli et al., 2017; Hu et al., 2019). This can be highly problematic under minimally invasive surgeries where the actuators would be introduced to wet conditions since these conditions would reduce the adhesion between CP electrodes and the SPE (Inoue et al., 2020). This delamination problem was solved by fabricating the device so that the CP electrodes and the SPE would entangled into a semi-interpenetrating polymer network (semi-IPN) (Vidal et al., 2003; Zhao et al., 2007; Thapliyal, 2010; Festin et al., 2011). These actuators did not delaminate, but they also required ionic liquid to function. Since ionic liquids are considered toxic, these actuators would be not be suitable in biological applications and so another method of fabrication is required (Zhao et al., 2007). Another study directly polymerized CP onto the Nafion, but this resulted in slow actuation responses (Khmelnitskiy et al., 2019). Using a high oxidant to CP monomer ratio, CP can polymerize onto the Nafion surface, but this leads to an uneven CP coating and a interfacial layer with a fixed thickness and shape (Tan and Bélanger, 2005; Pyshkina et al., 2019). Since this restricts the freedom in designing CP actuators another method of fabrication is desired.

**FIGURE 1:**
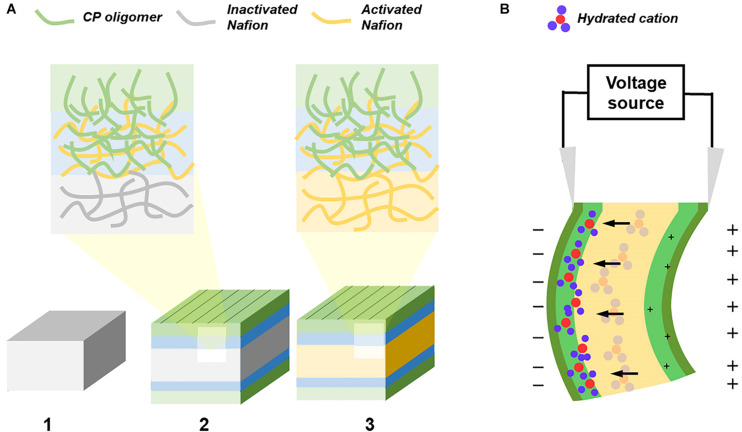
Graphical representation of the fabrication and actuation of CP/Nafion/CP actuator. **(A)** The fabrication of an ionic EAP with self-assembled electrodes starts with the (1) Nafion precursor pellets hot pressed into a membrane and its surface is then activated with NaOH while keeping is core still inactivated. The CP monomers polymerize and entangle itself into the activated Nafion polymer network while its interfacial layer acts as a seed for the CP shell layer. (2) The sample is then cut along the edges to allow for concentrated NaOH to (3) activate the entire Nafion without the CP oligomers diffusing into the core. **(B)** The ionic EAP is electrically stimulated and the hydrated cations move away from both the positively charged interfacial layer and the positively charged surface.

In this study, we present a facile technique of adding CP electrodes of either polyaniline (PANI) or poly(3,4-ethylenedioxythiophene) (PEDOT) onto a surface treated Nafion membrane through template-assisted self-assembly. The membrane is surface activated by treating precursor-Nafion membrane with NaOH which then converts its sulfonyl fluoride functional groups to sulfonate. Polymerizing the CP with the membrane allows us to fabricate an ionic liquid-free actuator that uses a semi-IPN of CP and Nafion-based SPE so that no delamination can occur (Figure 1). Using PEDOT electrodes, this actuator is biocompatible and so can be used in biological applications where a strong adhesion between layers is desired (Vreeland et al., 2015).

In a typical CP trilayer actuator, its electrodes are attached to the opposite sides of a fixed volume, ion-permeable membrane or to a SPE (Osada and Gong, 1998; Mirfakhrai et al., 2007; Hu et al., 2019). If the anions are mobile, applying a voltage would create a redox reaction at both electrodes. The hydrated anions would travel toward the oxidized electrode causing the oxidized side to swell and the reduced side to contract. In the cantilever configuration, the actuator would negatively deform and bend toward the cathode. In this study, the CP electrodes were attached to the Nafion-based SPE membrane (Figure 1B). Since the Nafion is negatively charged, the anions are immobile and so the hydrated cations would dictate the actuation and induce a positive deformation (Jager et al., 2000; Latonen et al., 2013).

As presented in Figure 1, CP electrodes self-assemble onto the surface treated precursor-Nafion membrane without the use of any adhesive or roughening and is bounded by a semi-IPN. The CP would penetrate into the interfacial layer of the membrane and is blocked from penetrating further due to the precursor-Nafion’s inactivated core. When the actuator is dried after each polymerization cycle, the Nafion membrane shrinks and exposes some of its CP from the interfacial layer to the surface which serves as a seed for the outer shell for self-assembly of the electrodes to occur (Xing et al., 2006).

Furthermore, the polymeric electrodes can self-assemble onto the Nafion membrane through ordered polymer growth and can be useful when adapting them into cost effective micro-actuators for neurosurgical applications.

## Materials and Methods

### Fabrication and Actuation of CP/Nafion/CP EAP

Nafion R-1100 precursor pellets (72500302, FuelCellStore) were hot pressed at 2000 psi using a benchtop hydraulic press (4386, Carver), to 180°C, for 5 min to form a membrane with an average thickness of 0.20 mm and 0.26 mm when the membrane is hydrated. The membrane was cut into 20 × 8 mm strips. Surface activation fabrication treatment (SAFT) consists of the Nafion precursor membrane being submersed into 0.1 M, 0.12 M, or 0.15 M NaOH at 90°C for 10, 30, 60, and 120 min in 2 mL centrifuge tubes in order to activate the surface of the precursor Nafion membrane. The membrane was washed twice with distilled water and then dried. For PANI/Nafion/PANI actuators, 1 mL of 0.2 M aniline in water and 1 mL of 0.25 M FeCl_3_ in ethanol (EtOH) (Venkatesh and Vishista, 2018) and the SAFT membrane was added into a 2 mL centrifuge tube. For consistency, the PEDOT/Nafion/PEDOT actuators’ concentrations of monomer and oxidant was the same as the actuators made with PANI, but 3,4-ethylenedioxythiophene (EDOT) was added into EtOH and FeCl_3_ was added into water. The polymerization reaction was left overnight for about 17 h. Once the reaction was completed and the CP diffused into the interfacial layer of the membrane, the membrane was dried with a Kimwipe and placed in a fresh 2 mL centrifuge tube to be dried. This step was done 3 times (cycles) to ensure the CP entirely coats the membrane. Afterward, the sides of the membrane were trimmed to roughly 15 mm × 4 mm to prevent short circuiting and was submerged in 1.25 M NaOH overnight to fully activate the core Nafion polymer which also is used to de-dope the CP electrodes (Li and Wan, 1998). To restore conductivity of the electrodes, the membrane was then re-doped by submerging the membrane in 0.01 M HCl overnight and then stored in 0.2 M acetic acid to reduce the change in pH of the sample solution. To measure the actuation performance, the EAP removed from storage solution, rinsed with distilled (DI) water, and dried with a Kimwipe. The EAP was then attached to a kelvin clip that was connected to a power supply (1688A, B&K Precision) and a 0.8 or 1.5 V was applied to actuate the device. The voltage was monitored by connecting a multimeter (EX330, Extech Instruments) to the power supply.

### Plotting the Ionic EAP’s Displacement for Kinematic Analysis

To compare actuators performances, one tip of the actuator was placed 4 mm into a kelvin clip at a vertical cantilever configuration. Its actuation was video recorded and then sent to a computer where its tip displacement was measured at every 1/3 of a second using Tracker (Ver. 5.1.2) software (Brown, 2013). Once the tip displacement was plotted, it was normalized with respect to the actuator’s gage length, which starts from the tip of the kelvin clip to the opposite end of the actuator (Nemat-Nasser, 2002). The tip displacement for PANI/Nafion/PANI actuator was fitted according to the following exponential function in order to quantitatively compare the response time, maximum displacement, and speed of the actuator at different SAFT conditions.

(1) $$\left(\frac{u}{\mathrm{Lg}}\right)=\mathrm{yoff}-A\,\left(1-\text{exp}\,\left(-\frac{t}{\tau }\right)\right)$$

Where u is the transverse tip deflection or the tip displacement in the x direction, Lg is the gage length which starts from the kelvin clip to the end of the of the EAP, A is the maximum normalized tip displacement, t is the time starting from when the voltage was applied, τ is the time constant but will also be defined here as the response time, and yoff is offset from the actuator’s starting position at *t* = 0 s. The offset is measured with respect to the tip of the kelvin clip in the x direction. There is an offset on the tested actuators since they were not entirely straight after fabrication and had a slight bent before actuation.

Previously, the response time was defined as the time when the actuation stopped (Nemat-Nasser and Wu, 2003), but since the tip displacement slows down exponentially, the exact time the actuation stops cannot be accurately determined. Instead, τ is found through regression analysis and is used to measure and compare the response time of the actuators across all samples.

We also reported Vmax, the maximum speed produced from the tip displacement, by finding the derivative of Eq. 1 and setting *t* = 0, to get the following equation:

(2) $$V\,\text{max}=\frac{A}{\tau }$$

Where A is the maximum change in normalized tip displacement, and τ as the response time.

The strain calculated for the actuator used the following formula (Cheedarala et al., 2014; Li et al., 2014; Nguyen et al., 2018).

(3) $$\varepsilon =\frac{2\,d\,\delta }{\left(L^{2}+\delta^{2}\right)}$$

Where ε is the strain, d is the thickness of the hydrated actuator, δ is the tip displacement, and L is the gage length of the hydrated actuator.

### Measuring Resistivity and Conductivity

The sheet resistance was measured using Keithley 2400 source meter with a 4-point probe. The resistance of the actuators can be converted into sheet resistance, Rs, by multiplying a correction factor using correlation tables (Smits, 1958).

(4) $$R\,s=\left(\frac{V}{I}\right)\,\left[C\,\left(s,a,d\right)\right]$$

Where s is the distance between each probe, a is the length, and d is the width of the EAP. The conductivity, κ, can then be found through the following equation:

(5) $$\kappa =\frac{1}{R\,s^{*}\,t}$$

Where t is the proposed conductive layer thickness that is assumed to be the outer and the interfacial layer of the dried EAP that was found through standard electron microscopy (SEM).

### Cross-Sectional Images of the EAP

The cross-sectional images of the EAP were captured using SEM and its elemental map was found using wavelength-dispersive X-ray spectroscopy (WDS). The actuators were frozen in liquid nitrogen and was fractured into two parts using a hammer. This method prevents distortion of the cross-sectional morphology caused by shear forces (Ferlita et al., 2008). SEM images which used secondary electrons (SE) were carried out using JEOL JSM-IT100 and images using backscattering electrons (BSE) in the composition (COMPO) mode were taken with JEOL JXA-8230 Electron Probe Microanalyzer.

## Results and Discussion

### PANI Fiber Growths Along the Outer Layer

**FIGURE 2:**
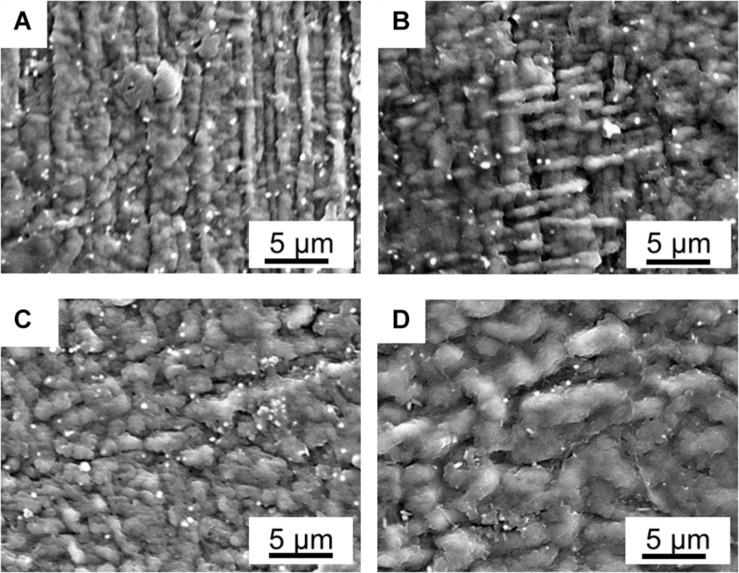
SEM pictures of fabricated PANI/Nafion/PANI actuators with a SAFT of 0.10 M NaOH for **(A)** 10 min, **(B)** 30 min, **(C)** 60 min, and **(D)** 120 min.

As shown in Figure 2, the SAFT duration of the precursor-Nafion membrane aid in the PANI electrodes’ characteristics and growth orientation. This is useful if a certain PANI growth orientation is desired. When the SAFT duration was 10 min, the PANI electrodes grew with fibril-like characteristics at a linear direction and when the duration was increased to 30 min, it grew at an orthogonal direction. From a SAFT duration greater than 60 min, the PANI polymerization growth became random. Previous literatures have also observed PANI nanofibers growing along surfaces (Huang and Kaner, 2004; Tan and Bélanger, 2005; Konyushenko et al., 2006), but none have yet shown that SAFT durations can be used to manipulate PANI’s growth orientation. The oriented growth of the polymer could be explained as a template-assisted self-assembly in that the SAFT alters the surface of the membrane in order to produce the desired growth orientation. Furthermore, polymerization seeding was observed in literature in aiding the formation of PANI nanofibers (Xing et al., 2006). This suggests that the PANI polymer network inside the interfacial layers could play a role in seeding the electrodes during polymerization.

### Sheet Resistance and Conductivity of the Fabricated EAP

Both PANI and PEDOT electrodes exhibited poor conductive properties as shown in its MΩ/sq sheet resistance values. This suggests that the high amount of sulfonate groups activated in the surface treated region may contribute to the CPs’ poor conductivity. Given that these sulfonate groups act as concentrated dopant anions, the PANI and PEDOT may have been polymerized with structural defects which would slow the rate of charges traveling through the CP and thus reducing its surface conductivity (Tehrani et al., 2007; Kim et al., 2016; Sakunpongpitiporn et al., 2019).

Since the PANI actuators’ measured sheet resistance was reduced after the first polymerization cycle, this suggests that the electrodes at the first cycle were not fully coated on the membrane surface. It was also observed that the PANI’s sheet resistance remained unchanged with respect to its SAFT durations. Provided that the high concentration of dopant decreases the CP’s conductivity, the amount of sulfonate groups on the treated surface must have been too high even with a SAFT duration of 10 min. The dopant concentration could be lowered by hydrating the membrane in acid before starting its polymerization cycles. This would convert some of the sulfonate groups to sulfonic acid. Some literatures have also suggested that the number of structural defects during polymerization can be reduced by lowering temperature of its reaction (Ayad and Rehab, 2008). They found that this slowed the polymerization growth rate which reduced the number of structural defects in the CP oligomers.

In PEDOT actuators, its sheet resistance remained unchanged with the increased number of polymerization cycles, but its sheet resistance decreased with longer treatment durations. Since the surface treatment duration of 10 min have fewer access to sulfonate dopants than longer surface treatments durations (Figure 4A), this suggests that the dopant concentration on the SPE surface at this treatment condition is not enough in producing saturated electrodes even after the third polymerization cycle.

**FIGURE 3:**
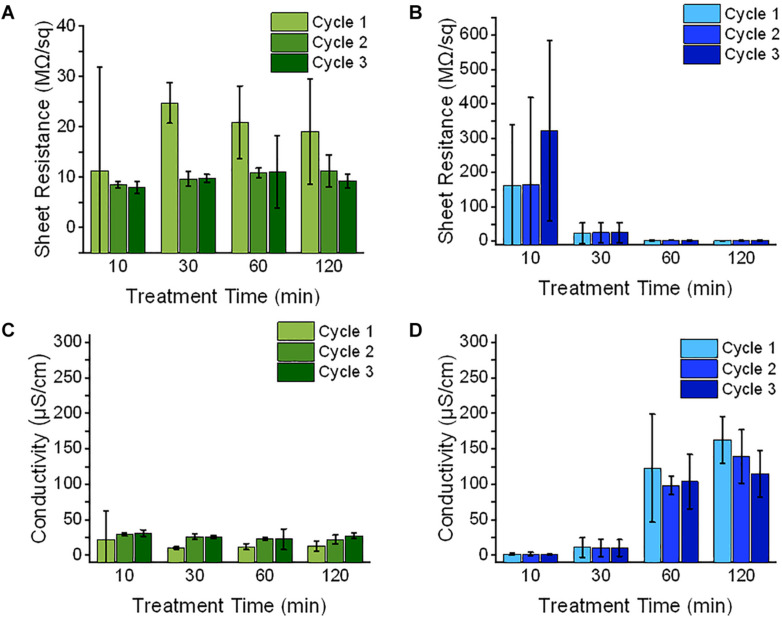
The electrical properties of EAP membrane (*n* = 3, SD) was measured during its fabrication with a 0.10 M NaOH SAFT with time durations of 10, 30, 60, and 120 min. **(A,B)** After each polymerization cycle of either PANI (green) or PEDOT (blue), the membrane’s sheet resistance was measured. The average thickness which includes of the shell and interfacial layers were measured using SEM images and **(C,D)** their conductivities were calculated.

Both PANI and PEDOT’s electrodes showed high variability at the first cycle. This can be observed by its high error bars as shown in Figures 3A,B, but during its third cycle, all the fabricated CP electrodes showed consistent measurements. This suggests that the CP oligomers were successfully coated on the surface treated membrane. The PANI polymerization cycles improved the EAP’s electrical properties and reduced its variation between samples.

**FIGURE 4:**
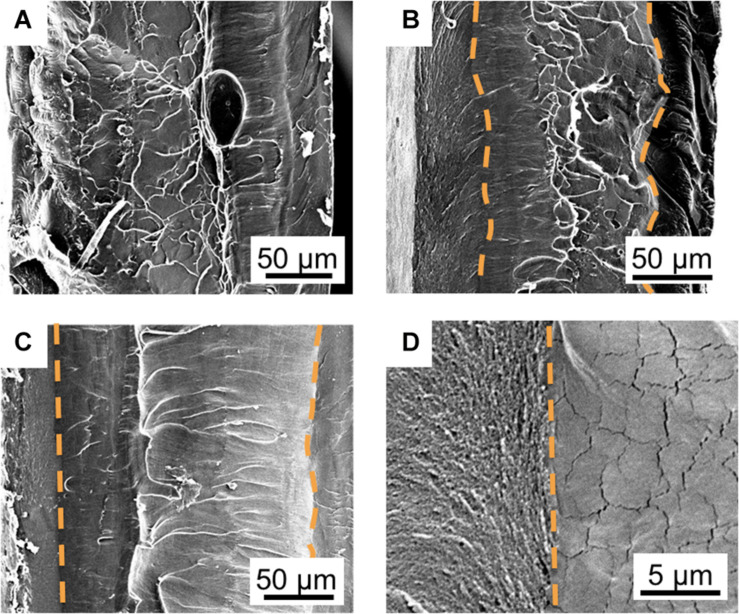
Controlling the shape of the interfacial and core layers through its SAFT duration. SEM cross-sectional images of PANI/Nafion/PANI actuators are shown in the vertical position with a SAFT concentration of 0.10 M NaOH at 90°C for **(A)** 10 min, **(B)** 30 min, **(C)** and 60 min. The orange dotted line follows the boundaries between **(D)** the interfacial (left) and core (right) layers which are determined by the differences in their morphologies.

The sheet resistance and conductivity were measured and calculated after every polymerization cycle. When the sheet resistance is converted into conductivity values using Eq. (5), the interfacial and outer layers of the EAP were assumed to be part of the conducting layer. The highest conductivity for the PANI actuators was 31.3 ± 4.5 μS/cm with a SAFT duration of 10 min. For PEDOT actuators, the highest conductivity recorded was 162.8 ± 32.6 μS/cm with a SAFT duration of 120 min. The PANI/Nafion and PEDOT/Nafion composites was reported to have conductivities several orders of magnitude higher with values than this study at 0.010 S cm^–1^ and 3 S cm^–1^, respectively (Hsu, 1991; Simotwo and Kalra, 2016; Hofmann et al., 2020). The lower than reported conductivity measurements found in this study may suggest that the interfacial layers were not conductive but instead merely acts as a pseudocapacitive layer (Kim et al., 2010; Huang et al., 2014).

### Controlling the PANI/Nafion Interfacial Layer

The boundary between the interfacial and the core layer became more defined as the SAFT duration increased (Figure 4). At 10 min, the two layers indistinguishable, but at 30 min, the interfacial layer showed a coarse grain structure and the core layer showed a smooth structure with microcracks. The two layers were separated by a wavy boundary line, but at a SAFT duration of 60 min, a straight boundary line was observed. Using SAFT, the interfacial and core layers can be easily fabricated to produce a wavy or straight design without the use of a custom mold or a milling machine. The interfacial layer’s shape and thickness can controlled by changing the activated fabrication treatment (SAFT) conditions. This can then act as a pseudocapacitive layer which has shown that increasing the actuator’s capacitance, they were able to improve its performances (Cheedarala et al., 2014; Kotal et al., 2016).

**FIGURE 5:**
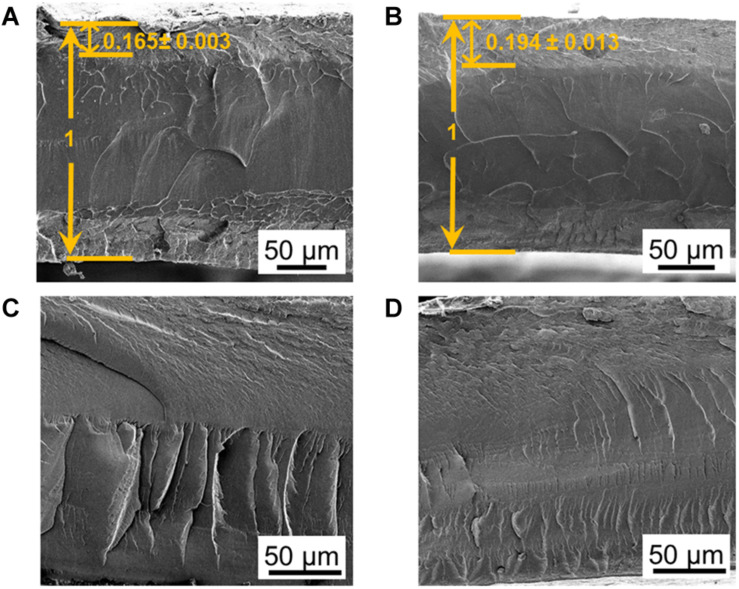
Controlling the thickness of the interfacial layers through its SAFT concentration. SEM cross-sectional images of PANI/Nafion/PANI actuators are shown in the horizontal position with SAFT concentrations of **(A)** 0.10 M NaOH, **(B)** 0.15 M NaOH, **(C)** 0.01 M NaOH, **(D)** and 1.25 M NaOH. The interfacial thicknesses measured were normalized with respect to its actuator’s overall thickness.

When increasing the SAFT concentration of NaOH from 0.10 to 0.15 M, the interfacial layer became thicker, from a normalized thickness of 0.165 ± 0.003 to 0.194 ± 0.003 (*n* = 2, SD) when compared to its overall thickness (Figure 5). This suggests that interfacial layer can be controlled by changing the NaOH concentration during the fabrication process. The electrodes and the penetration depth can be designed at the 0.10 M NaOH range to maintain its consistency. When using low concentration of NaOH such as 0.01 M, this can cause the interfacial thickness to become wavy and uneven (Figure 5C), and at higher NaOH concentrations such as 1.25 M (Figure 5D), this causes the CP oligomers to penetrate to the center of the Nafion core.

**FIGURE 6:**
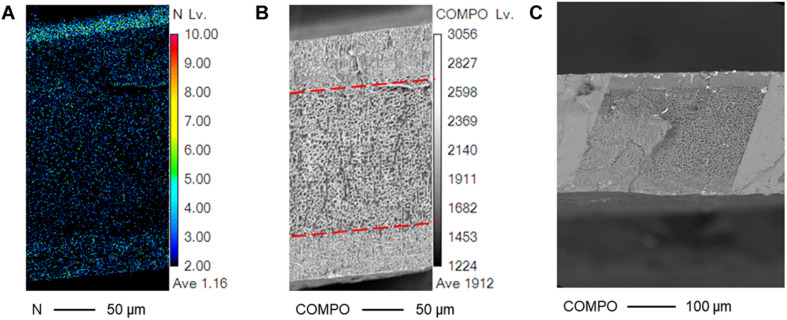
Nitrogen and COMPO mapping of a PANI/Nafion/PANI actuator. SEM cross-sectional pictures of the actuator are shown with a SAFT concentration of 0.10 M NaOH for 1 h using **(A)** WDS and **(B,C)** BSE.

The morphology between the interfacial and the core layer becomes more apparent after observing its nitrogen elemental map and its COMPO map (Figure 6). The nitrogen elemental map showed dense nitrogen levels in the interfacial layers. Provided that only the PANI polymer network and not the Nafion membrane is composed of nitrogen, this verifies that the PANI network from the membrane surface reached into the interfacial layers of the membrane. This also suggests that the actuator’s interfacial layer is a form of a IPN between the CP and the SPE network. The CP in the interfacial layer interlocked with the SPE through a semi-IPN and so the two networks can only be separated by breaking its bonds at a molecular level and thus would be viable in bio applications where no delamination is desired (Vidal et al., 2003; Thapliyal, 2010; Inoue et al., 2020).

The sparse nitrogen density around the core polymer in Figure 6 suggests that the PANI network barely reached into the core polymer during fabrication. Furthermore, its nitrogen density is also not as high on the bottom surface as compared to its top. This might suggest an uneven IPN, but the symmetrical cross-sectional damage observed through the COMPO map may suggest otherwise. Observing the COMPO map, the interfacial layer remained primarily undamaged by the electron beam while the core layer appeared porous and sponge-like. Previous literatures observed that Nafion and other organic membranes become damaged under SEM, but they also noted that when the Nafion contained positive heavy counterions, the damage was minimal (Yakovlev et al., 2013; Leijten et al., 2017). This could explain for the actuator’s symmetrical damage caused by SEM. The PANI oligomers protected the actuators’ interfacial layer and so only the core Nafion layer was mostly damaged. The nitrogen elemental map could have been quantitatively inaccurate due to its rough cross-sectional surface caused the freeze fracture procedure and due to the slight tilt occurred when placing the membrane on the SEM specimen stub. Since the nitrogen elemental map was performed using WDS, this technique is sensitive to slight angle variations in that rough cross-sectional surfaces could cause noticeable quantitative errors (Newbury and Ritchie, 2014).

**FIGURE 7:**
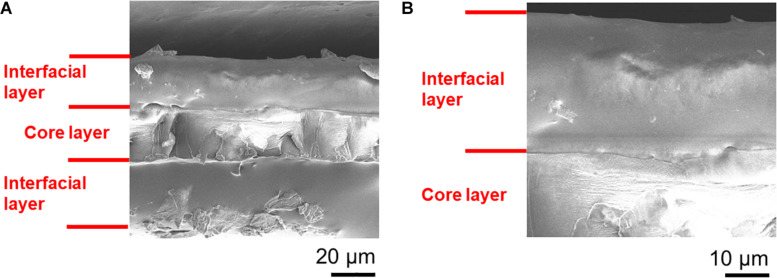
The swelling of the interfacial layers in a hydrated PANI/Nafion/PANI actuator. SEM images shown are of **(A)** its overall cross-section and **(B)** its close-up. Images were taken immediately after gold sputtering in order reduce the dehydration of the actuator.

In the SEM and WDS images shown in Figures 5, 6, the cross-sectional morphology and nitrogen map differs between the outer, interfacial, and core layer. This suggests that these are separate layers, and this is further verified in Figure 7. The hydrated PANI/Nafion/PANI actuator was put into the SEM where it captured images of the swollen interfacial layers. This suggests that the interfacial layers is able to pull water from the core layer and allowing it to participate in the actuations.

### Actuation Studies for all Polymeric Ionic EAP

**FIGURE 8:**
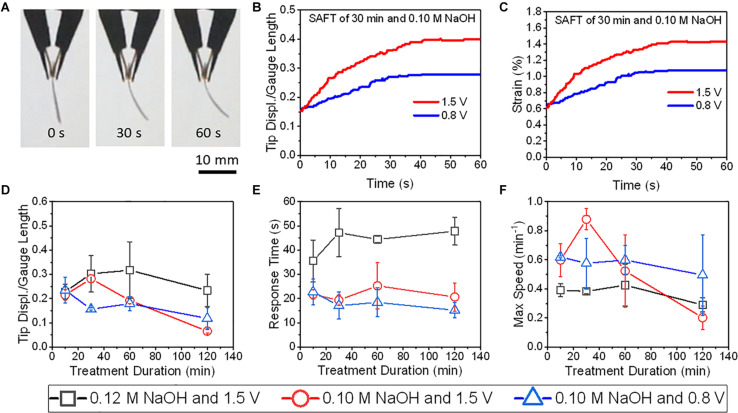
The actuations of PANI/Nafion/PANI actuators at various applied DC voltages and SAFT conditions. **(A)** Shows the time-lapse images of an actuator with a SAFT duration of 30 min actuated at 1.5 V. **(B)** The actuators’ normalized tip displacements with respect to time. **(C)** The actuators’ strains with respect to time. **(D)** The actuators’ normalized tip displacements with respect to SAFT duration. **(E)** The actuators’ response times with respect to SAFT duration. **(F)** The actuators’ max speeds with respect to SAFT duration (*n* = 2, SD).

The actuation performance with PANI electrodes were compared at 0.80 V and at 1.5 V and found that only actuators with SAFT duration of 30 min did the tip displacement and maximum speed improved with voltage (Figure 8). Other than actuators with 10 min SAFT durations, the response time improved with increased voltage. The PANI actuators with a SAFT concentration of 0.12 M NaOH had higher tip displacements than actuators with a SAFT concentration of 0.10 M NaOH but they also experienced a slower response and a smaller maximum speed. Actuators with 30 min SAFT durations the best out of all durations where it displayed higher tip displacements, a quicker response, and greater maximum speeds. This suggests that the interfacial layer plays a role in the actuator’s performance. A SAFT duration of 30 min produced a wavy boundary line between the interfacial and core layers which increases the surface area between the two layers (Figure 4). This would increase the rate of water molecules being transferred between the layers. When actuated at 1.5 V, the actuator had a far greater maximum speed than at 0.8 V. This suggests that the redox reaction is important in the actuator’s performance. Both the boundary layer shape and the voltage applied was shown to improve the actuator’s maximum speed. Furthermore, by increasing the SAFT duration from 30 min, the actuator’s performance worsened. Longer SAFT durations resulted in a straighter boundary line between the layers, and as a consequence, reduced the surface area between the interfacial and core layer and its actuation performance.

**FIGURE 9:**
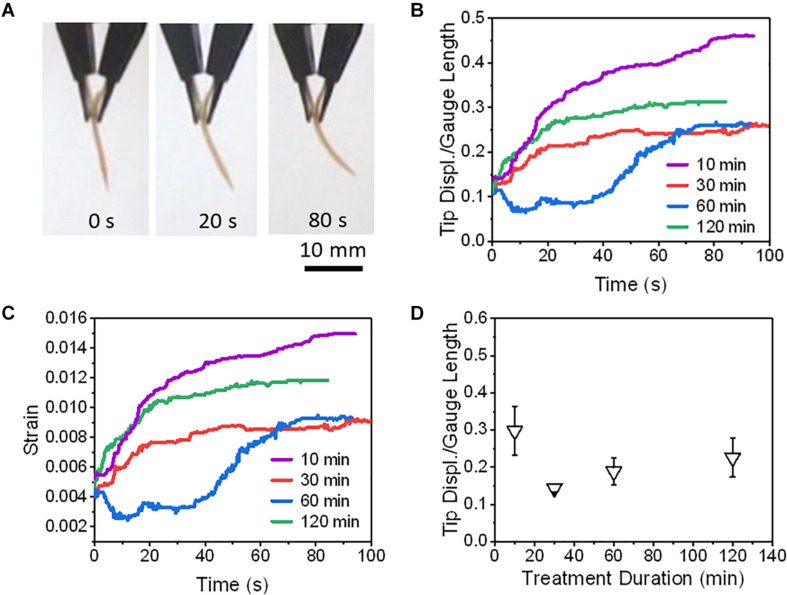
The actuations of PEDOT/Nafion/PEDOT actuators at different fabrication conditions. Panel **(A)** shows the time-lapse images of an actuator that was actuated at 1.5 V and was fabricated with a SAFT duration of 10 min. Actuators at different SAFT times were actuated at 1.5 V to compare their **(B)** normalized tip displacements, and **(C)** strains with respect to time. **(D)** Actuators were actuated at 1.5 V and their normalized tip displacements were compared with respect to SAFT duration (*n* = 2, SD).

The highest deformation recorded in this study was a PEDOT/Nafion/PEDOT actuator with a SAFT duration of 10 min. At 1.5 V, its normalized tip displacement was recorded to be 0.32 and applying Eq. (3), its overall strain was found to be 1.0% (Figure 9). When compared to PANI/Nafion/PANI actuators with the same SAFT and voltage conditions, it displayed a lower normalized tip displacement of 0.22 with a maximum strain of 0.6% (Figure 8). At a SAFT duration of 30 min, PANI/Nafion/PANI actuators displayed its highest tip displacement and strain of 0.25 and 0.8%, respectively. Others in literature who have attempted to use CP electrodes for their actuators experienced strains of around 0.4% when actuating less than 2 V (Lee et al., 2013; Cheedarala et al., 2014; Li et al., 2014; Rasouli et al., 2017). And in another similar study, they directly polymerized PANI and PEDOT onto a manually roughened Nafion membrane without any SAFT (Khmelnitskiy et al., 2019). They were able to achieve higher maximum strains at 1.0 V than the SAFT actuators used in this study, but its response time was reported to be greater than 9 min (Khmelnitskiy et al., 2019). When comparing to SAFT actuators that were held at 1.5 V, many of the actuators finish deforming in under a minute (Figures 8, 9). SAFT actuators has a shorter response due to the improvements in the interfacial layer’s design where it acts as a better pseudocapacitor. And by improving the capacitance in actuators, its actuation performances also improved (Cheedarala et al., 2014; Kotal et al., 2016),

**FIGURE 10:**
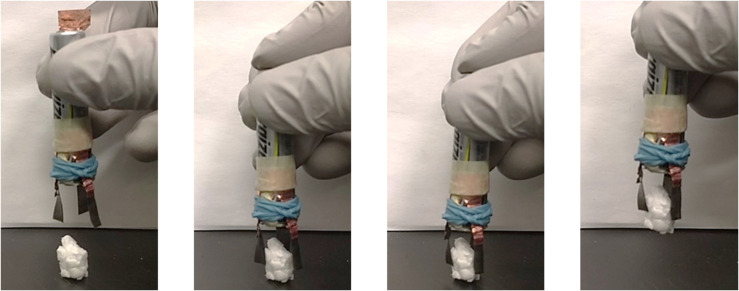
Demonstration of a PANI/Nafion/PANI gripper picking up an 88 mg Styrofoam block (see Supplementary Video S1). The gripper used a AAA 1.5 V battery to actuate.

Some of the PEDOT/Nafion/PEDOT actuators showed negative deformation at the beginning of actuation but they overall deformed in the positive direction (Figure 9B). The tip displacement with respect to time also did not follow the exponential equation shown in Eq. (1) and so its response time and maximum speed was not calculated. The negative deformation could be due to the redox reaction that occurs in the interfacial and outer layers during actuation. The charged PEDOT at the cathode end would become reduced while the neutral PEDOT at the anode end would become oxidized. Once reduced, the PEDOT would lose its charge and the water molecules would be free to diffuse back into the Nafion core. At the same time, the oxidized PEDOT would become charged and the water molecules would get drawn toward the anode causing a negative deformation in the actuator.

### Potential Practical Application

To show the practical application of proposed actuation system, a gripper was assembled using the fabricated PANI/Nafion/PANI actuator. The three fabricated actuators were fixed onto a 1.5 V AAA battery circumferentially, as shown in Figure 10. To activate the gripper, the copper switch was added on the negative terminal. The simultaneous activation of each actuator from the same external source, but in varying directions, causes the device to achieve a gripping motion. This allowed the device to successfully pick up an 88 mg Styrofoam block within 1 min. A traditional design for a surgical gripper that is used in minimal invasive neurosurgical tools is 5 mm and to retract certain portions of the brain is approximately 80 mN (Chen et al., 2020). In this design, the gripper shown is able to lift an 88 mg styrofoam block or 0.86 N and so it is capable of retracting certain portions of the brain. The gripper in this application is roughly twice the size of graspers found in minimal invasive neurosurgical tools. Since the electrodes are made from CPs, it is possible to miniaturize this device and can be produced at a fraction of the cost. The disadvantage of applying this tool to neurological surgery would be the long response time. Considering that that the limit of acceptable time delay between actuating a tool and observing its visual stimulus was found to be 330 ms before the surgeon is risking their perceived safety for the patient (Marescaux et al., 2001), further studies will be required to improve the response time of the actuator. Since the electrodes were shown to have poor electrical properties, we might be able to improve its response time by improving the conductivity of the electrodes.

## Conclusion

Previous studies replaced metallic electrodes with CP for cheaper fabrication of EAPs, but these devices generally experience little to no noticeable strains at lower voltages (<2.0 V). Oftentimes, these devices require lamination during fabrication and so is prone to wear and tear.

In our study we were able to improve the production, performance, and durability by growing polymeric electrodes directly onto a surface treated Nafion membrane. This novel process resulted in the production of an actuator with a core shell polymer design and controllable pseudocapacitive layers. The layers are bounded by semi-IPN between the CP electrode and the Nafion membrane and both of which can be biocompatible and will never delaminate. We utilized SAFT at different concentrations and treatment durations which allowed for the freedom to design the actuator’s interfacial layer, in thickness and in consistency and also the freedom to control the CP’s polymerization growth orientation along SPE surface which can help to optimize the actuator’s performance.

Our prototype demonstrates controllable layers due to it’s self-assembly behavior and is more cost effective than metallic EAP actuators. This translates into a favorable scalability for large manufacturing due to the facile nature of this technique.

## Data Availability Statement

The raw data supporting the conclusions of this article will be made available by the authors, without undue reservation.

## Author Contributions

AJ contributed to the methodology, the data curation and processing, and the manuscript writing. CH has helped with the data collection. HN has supervised and guided the research in it’s the methodology, and the development of the manuscript. All authors contributed to the article and approved the submitted version.

## Conflict of Interest

The authors declare that the research was conducted in the absence of any commercial or financial relationships that could be construed as a potential conflict of interest.

## Supplementary Material

VIDEO S1 Video that shows the PANI/Nafion/PANI Actuator gripping an 88 mg Styrofoam block.
